# Low-False-Alarm-Rate Timing and Duration Estimation of Noisy Frequency Agile Signal by Image Homogeneous Detection and Morphological Signature Matching Schemes

**DOI:** 10.3390/s23042094

**Published:** 2023-02-13

**Authors:** Yuan-Pin Cheng, Chia-Hsuan Chang, Jung-Chih Chen

**Affiliations:** 1 Electronic Systems Research Division, National Chung-Shang Institute of Technology, Taoyuan City 335, Taiwan; 2 College of Electrical and Computer Engineering, National Yang-Ming-Chiao-Tung University, Hsinchu City 300, Taiwan; 3 Institute of Biomedical Engineering, National Yang-Ming-Chiao-Tung University, Hsinchu City 300, Taiwan

**Keywords:** FHSS detection, morphological matching process, dual-diagonal operator, diagonal edge finding, temporal correlation function, image erosion, median filter, discrete wavelet transfer, Hough transform

## Abstract

Frequency hopping spread spectrum (FHSS) applies widely to communication and radar systems to ensure communication information and channel signal quality by tuning frequency within a wide frequency range in a random sequence. An efficient signal processing scheme to resolve the timing and duration signature from an FHSS signal provides crucial information for signal detection and radio band management purposes. In this research, hopping time was first identified by a two-dimensional temporal correlation function (TCF). The timing information was shown at TCF phase discontinuities. To enhance and resolve the timing signature of TCF in a noisy environment, three stages of signature enhancement and morphological matching processes were applied: first, computing the TCF of the FHSS signal and enhancing discontinuities via wavelet transform; second, a dual-diagonal edge finding scheme to extract the timing pattern signature and eliminate mismatching distortion morphologically; finally, Hough transform resolved the agile frequency timing from purified line segments. A grand-scale Monte Carlo simulation of the FHSS signals with additive white Gaussian noise was carried out in the research. The results demonstrated reliable hopping time estimation obtained in SNR at 0 dB and above, with a minimal false detection rate of 1.79%, while the prior related research had an unattended false detection rate of up to 35.29% in such a noisy environment.

## 1. Introduction

Frequency hopping spread spectrum (FHSS) is one of the broadly used electronic countermeasures (ECM) of spread spectrum modulation technology applied on communication and radar systems to ensure communication information and channel signal quality. The scheme is well suited for transmission in the presence of noisy distortions or jamming deceptive environments. FH is a technique in which the carrier frequency jumps following a pre-determined hopping pattern (HP) only designated and acknowledged by the transmitter and receiver sides. In the electronic counter-countermeasure ECCM perspective, an efficient signal processing scheme to extract the timing signature from the FH signal provides crucial information to resolve the signal frequency out of this unknown frequency agile varying sequence in this hopping duration.

Target FH signals in the real world are commonly received under influence of heavy noisy signals, resulting in low signal-to-noise ratios. This research proposed a series of morphologically enhanced de-noise processes to match the signature of TCF cross-term trajectory to develop an efficient and robust FH signal hopping time detection scheme.

**Figure 1:**
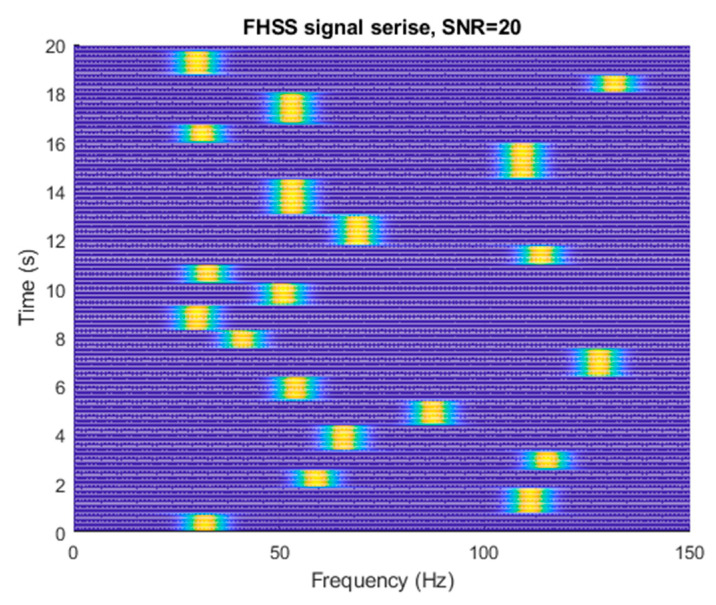
Frequency-hopped signal, twenty frequency hops, sampling frequency equal to 250 Hz.

Time of frequency change and frequency used are essential information in hopping patterns. The receiver records and analyzes the hopping patterns and then recovers the information or message [1]. It is not suitable to only use a Fourier transform method to analyze an FH signal when the hopping time has a jitter because the information on hopping time may be averaged by the sampling window [2]. This study proved discontinuities of signals presented between two frequencies. Overdyk investigated detection frequency hopping time locations in frequency-hopped (FH) schemes via temporal correlation function (TCF), which examined the cross-term and wavelet processes in one-dimensional data analysis [3]. The FHSS schemes were designed for digital information transmission in which binary data bits were divided into blocks of a fixed size. Each block represented a unique carrier frequency that was sent through the channel, called a symbol [4]. Therefore, an FH signal was composed of a series of narrowband tones with different frequencies, which was defined as a uniform random variable in the range of the available bandwidth, and N was the number of available frequency hops. Figure 1 presents a series of illustrative FH signals with twenty frequency hops, where the signal frequencies were statistically uncorrelated and uniformly distributed over the bandwidth of 80 Hz.

The existing FH detection research analyzing signal discontinuity through one-dimensional TCF information focused on pursuing a high detection rate, while false detection control was not well considered. The drawback was growing exponentially in a noise-intensity environment, and the false alarm rate was over 35% in SNR = 0 dB [5,6]. The intolerable false detection rate severely deteriorated the reliability of the current detection methodologies. This research analyzed TCF phase term in two-dimensional (2D) quantity and took advantage of the diagonal line-up trajectory of the cross term over time lagging indices. Two-dimensional morphological analysis of TCF was proposed later in FH detection [7] and phase-shift-key (PSK) signal detection application [8]. Nevertheless, column-wise and row-wise aligned kernel edge operators were applied in this research and mismatched oriented kernels were demonstrated to deteriorate detection rate in 2D TCF morphological analysis in this study.

To enhance the specific character of a ±45-degree diagonal timing signature in the TCF, a V-shaped dual-diagonal operator (DDO) for matched pattern operation was investigated in this research. Based on the concept of edge detection, this novel DDO kernel was applied to gradient calculation [9] in this study.

An innovative image processing approach and homogeneous analysis were applied to enhance TCF diagonal trajectory and suppress the disorienting effect of noise distortion. Through these morphological matching processes, the timing signature of the FHSS series was resolved successfully at a high detection rate, 98.2%, with a minimal false alarm rate, which decreased to 1.79% in SNR at 0 dB and above. The scheme performed more than 62% probability of detection as SNR decreased to −3 dB, while false alarm still managed to maintain a similar level as in higher SNR.

## 2. Materials and Methods

To identify the hopping timing signature of the FHSS signal, a series of signal enhancement and denoise schemes were applied and initialized from the cross-term trajectory of a time-spatial correlation function called temporal correlation function (TCF). The TCF analyzed the cross correlation of the signal in terms of time lags with phase indicating the frequency deviations.

### Temporal Correlation Function (TCF)

This section introduced the concept and definition of TCF and how the phase term of the cross correlation revealed the initial timing signature out of a frequency agile signal. The two-dimensional TCF results were followed up by morphological processes to accomplish the frequency hopping resolving schemes.

The temporal correlation function (TCF) of a signal *x*(*t*) is defined as [3] (p. 47)

(1) $$TCF_{x}(t,\tau )=x\left(t+\frac{\tau }{2}\right)\cdot x^{*}\left(t-\frac{\tau }{2}\right),$$

where *t* is the signal time index and *τ* is the time lagging index of the signal. In this research, the TCF phase matrix was applied to the detection algorithm.

#### TCF Definition

Let *x*(*t*) be defined as the non-stationary frequency hopping analytic signal:

(2) $$x_{a}(t)=e^{i2\pi f_{1}t}\left[u(t)-u(t-T_{hop})\right]+e^{i2\pi f_{2}t}\left[u(t-T_{hop}+1)-u(t-T)\right],\;0\leq t\leq T,$$

where *T_hop_* is the hopping time at which point the message frequency hops from frequency *f*_1_ to *f*_2_, and *u*(*t*) is the unit step function defined as:

(3) $$u(t)=\{\begin{array}{cc} 1 & \;,\mathrm{for}\;t\geq 0\\ 0 & ,\mathrm{for}\;t\text{<}0 \end{array}$$

Substituting Equation (2) into Equation (1) leads to [3] (p. 49):

(4) $$\begin{array}{l} TCF(t,\tau )=\{\begin{array}{c} e^{i2\pi f_{1}\tau }\left[\begin{array}{l} u\left(t+\frac{\tau }{2}\right)\left[u\left(t-\frac{\tau }{2}\right)-u\left(t-\frac{\tau }{2}-T_{hop}\right)\right]\\ +u\left(t+\frac{\tau }{2}-T_{hop}\right)\left[u\left(t-\frac{\tau }{2}-T_{hop}\right)-u\left(t-\frac{\tau }{2}\right)\right] \end{array}\right]\\ +e^{i2\pi f_{2}\tau }\left[\begin{array}{l} u\left(t+\frac{\tau }{2}-T_{hop}+1\right)\left[u\left(t-\frac{\tau }{2}-T_{hop}+1\right)-u\left(t-\frac{\tau }{2}-T\right)\right]\\ +u\left(t+\frac{\tau }{2}-T\right)\left[u\left(t-\frac{\tau }{2}-T\right)-u\left(t-\frac{\tau }{2}-T_{hop}+1\right)\right] \end{array}\right]\\ e^{i2\pi \left[(f_{2-}f_{1})t+\left(\frac{f_{1}+f_{2}}{2}\right)\tau \right]}\left[\begin{array}{l} u\left(t+\frac{\tau }{2}-T_{hop}+1\right)\left[u\left(t-\frac{\tau }{2}\right)-u\left(t-\frac{\tau }{2}-T_{hop}\right)\right]\\ +u\left(t+\frac{\tau }{2}-T\right)\left[u\left(t-\frac{\tau }{2}-T_{hop}\right)-u\left(t-\frac{\tau }{2}\right)\right] \end{array}\right] \end{array}\\ =TCF_{1}(t,\tau )+TCF_{2}(t,\tau )+TCF_{12}(t,\tau ). \end{array}$$

**Figure 2:**
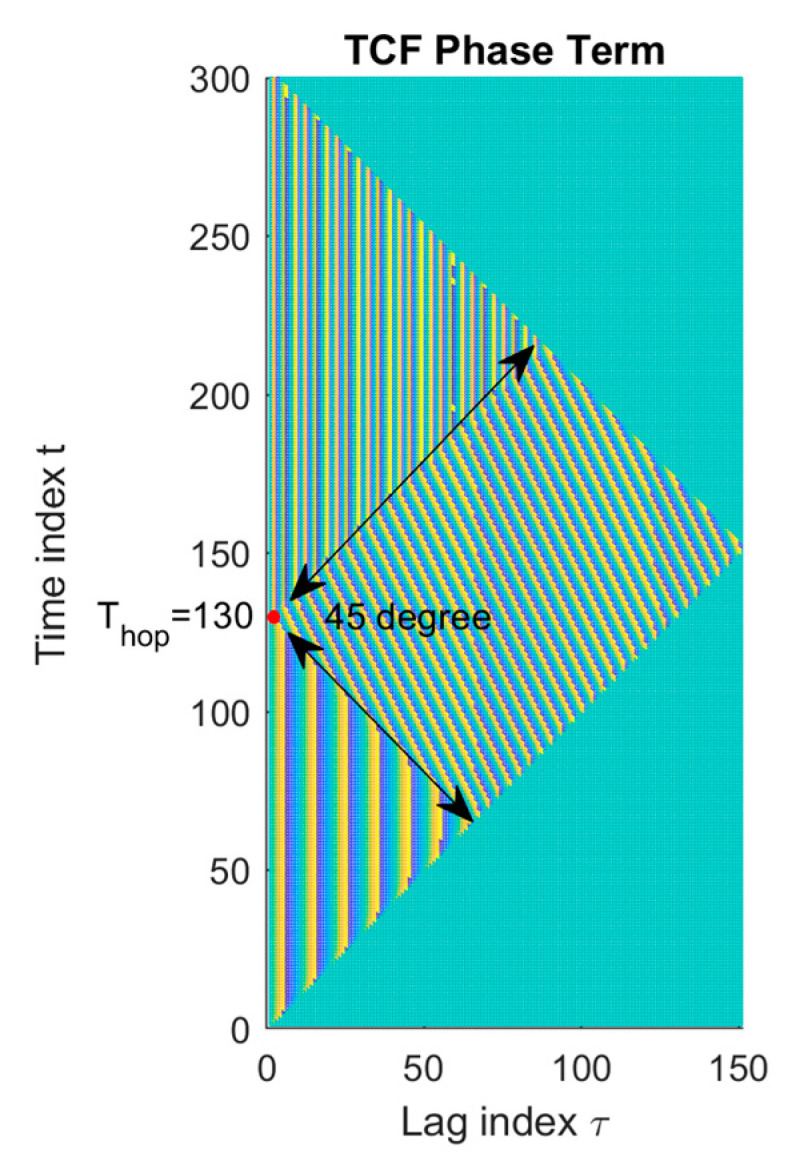
TCF angle of a non-stationary analytic frequency hopping signal; hopping time location at t = 130. Adapted with permission from Ref. [7]. Copyright 2008, Y-.P. Cheng

Terms *TCF*_1_(*t*,*τ*), *TCF*_2_(*t*,*τ*), and *TCF*_12_(*t*,*τ*), respectively, for the corresponding 1st, 2nd, and 3rd terms included in Equation (4). The unit step expressions confined the boundaries of these three terms and formed non-overlapping regions that composed a complete TCF expression. Note that the phase of the *TCF*_1_(*t*,*τ*) and *TCF*_2_(*t*,*τ*) terms expressed as a function of the variable “t” are constant and equal to 2*πf*_1_*τ* and 2*πf*_2_*τ*, respectively, while the phase contribution contained in term *TCF*_12_(*t*,*τ*) varies in terms of both variables *t* and *τ*. The phase expression of three terms form the boundaries in a shape of equilateral triangle, as shown in Figure 2. The TCF phase expressed as a function of “t” for a time difference “τ” and exhibited the change in phase continuity between auto terms and cross terms.

Therefore, the TCF phase expressed as a function of “t” for a time difference “τ” exhibits change in phase continuity between auto terms and cross terms. Figure 2 illustrates the behavior of the phase of the TCF matrix computed from the signal *x_a_*(*t*) in Equation (2), where *f*_1_ = 15 Hz, *f*_2_ = 45 Hz the hopping time *T_hop_* = 130, and the total signal duration *T* = 300, which was also the experimentally frequency-agile-signal design used in the study. Figure 2 showed that the hopping time signature was revealed at the left vertex of the triangle trajectory of *TCF*_12_(*t*,*τ*) region and the instant *T_hop_* could resolve by indicating the intercepted time index from the two diagonal lines of the boundary in *TCF*_12_(*t*,*τ*). The boundary of cross term *TCF*_12_(*t*,*τ*) held ±45° orientated lines between two auto terms of *TCF*_1_(*t*,*τ*) and *TCF*_2_(*t*,*τ*).

The changes in TCF phase as a function of “t” are computed to automatically detect edges for resolving *T_hop_*. The process contains a one-dimensional wavelet transform that emphasizes discontinuous edges and a two-dimensional dual-diagonal operator (DDO) for morphological matched filtering operations. The timing signature enhancement and morphological operation used in this study were introduced in Section 3 and Section 4.

**Figure 3:**
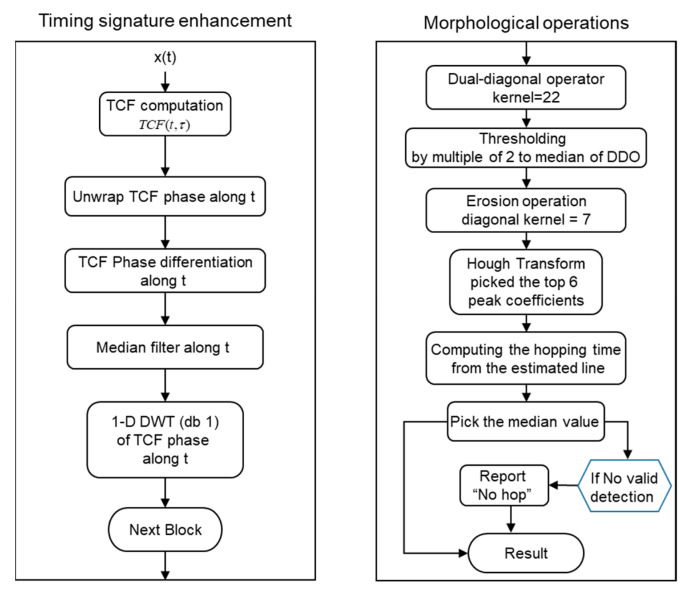
Overall FHSS signal timing information estimation scheme flow chart.

The FHSS timing detection and estimation scheme was processed by two main phases, illustrated in Figure 3.

Phase 1: Timing signature enhancement applying to the region boundaries of TCF phase terms.

Phase 2: Applying morphological operations in edge finding and denoise proceed by matched oriented kernel operation and resolving timing information based on binary images.

## 3. Timing Signature Enhancement

**Figure 4:**
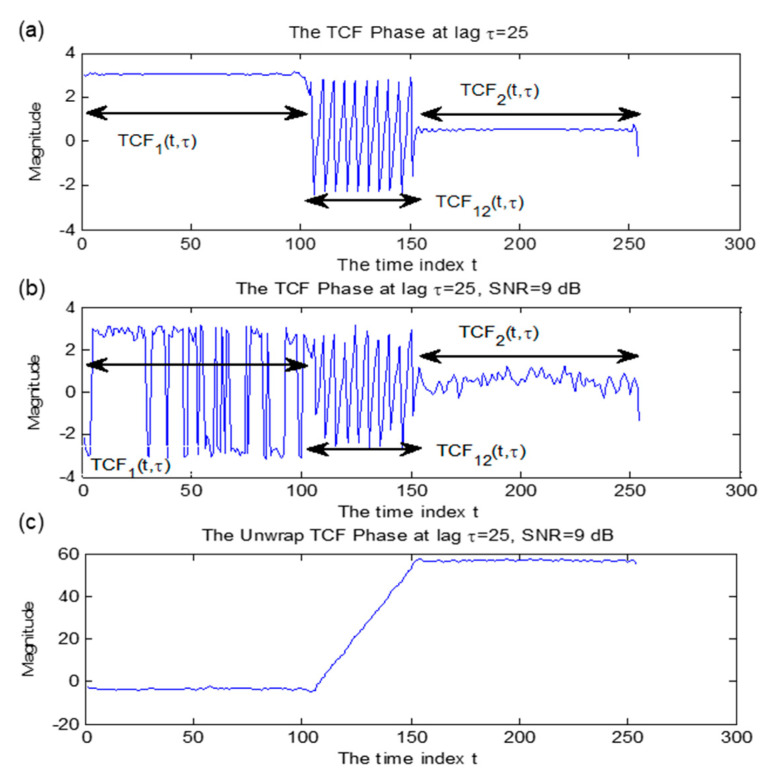
One−dimensional TCF phase analysis of a non−stationary analytic frequency hopping signal at τ = 25. Hopping timing signature captured at cross term *TCF*_12_, while auto−term *TCF*_1_ and *TCF*_2_ represent static frequency analysis. In (**a**), a noise−free non−stationary analytic frequency hopping signal was presented, while (**b**) shows the noise distortion in phase term of TCF with SNR = 9 dB; (**c**) was the unwrapped phase term of (**b**) to obtain the continuous phase expression of TCF.

The TCF phase matrix exhibited hopping time locations in a noise-free environment, which was the baseline timing signature information of hopping time in this study, and a TCF phase vector at τ = 25 calculated from a noise-free frequency agile signal was shown in Figure 4a. The phases of auto-term *TCF*_1_ and *TCF*_2_ were distinct from the phase of cross term *TCF*_12_. However, the timing signature depicted by the trajectories of the cross term and auto term degraded significantly as noise interference increased, as in Figure 4b, the phase of TCF in FH signal distorted by additive white Gaussian Noise (AWGN) with SNR = 9 dB for lag τ = 25. This example showed that the hopping time information was not exposed regularly as the noise introduced discontinuous phase information. Thus, additional processing becomes necessary to increase the robustness of the detection scheme. This section discusses three pre-processing steps:

(1) unwrapping the phase information;

(2) differentiating the unwrapped phase to emphasize discontinuous trajectories of the phase;

(3) applying a median filter to reduce noise effects.

### 3.1. Phase Unwrapping Function

Note that phase values of *TCF*_1_ in Figure 4b were jumping vigorously around π and -π as the phase error had been introduced by noise and failed to represent the true analytical phase of the signal. Unwrapping transforms jump larger than *π* between successive points to their 2*π* complement. The TCF phase, *p*(*t*), of the signal, *x*(*t*), may be unwrapped as follows [6].

(5) $$unwrap\left(p(t)\right)=\{\begin{array}{cc} p(t) & ,if\;\left|p(t)-p(t-1)\right|\leq \pi \\ p(t)+2\pi & ,if\;p(t)-p(t-1)<-\pi \\ p(t)-2\pi & ,if\;p(t)-p(t-1)>\pi \;. \end{array}$$

Regarding the result shown in Figure 4c, the unwrapped phase of TCF maintained constant in the auto-term *TCF*_1_ and *TCF*_2_ and presented a monotonic increment in cross term *TCF*_12_. The phase behavior was expected to be linear in the *TCF*_12_ region (when expressed as a function of t for a fixed τ value) for times in the range [100, 160]. The result showed that the slope of the *TCF*_12_ region was linear, as expected. After the unwrapping procedure, the plot exhibited a large step value between *TCF*_1_ and *TCF*_2_ regions to ease the impact of relatively small phase variation out of noise distortion.

### 3.2. Differentiation

**Figure 5:**
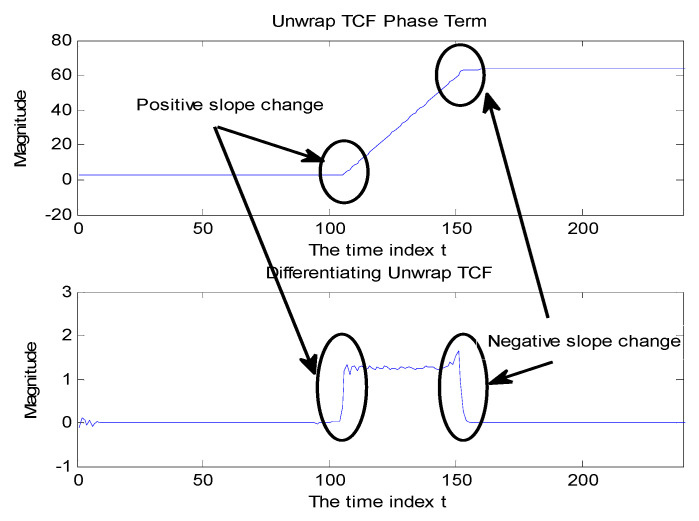
The top plot showed the unwrapped TCF phase with a positive slope in the cross term in t at [100, 160]. The bottom plot was differentiation applied to the unwrapped TCF phase along the time axis in constant τ to indicate positive and negative slope difference at t = 100 and 160, respectively.

Holding τ constant, the expected TCF phase behavior expressed as a function of t is a succession of three stages: flat stages in the *TCF*_1_ and *TCF*_2_ regions, while *TCF*_12_ region is a ramp. This situation represents the discontinuity in the phase expressed as a function of t (while keeping τ fixed). The discontinuity situations were emphasized by differentiating the phase along the time axis. The differentiation operation calculates the difference between adjacent points [10]. The result of differentiation of the unwrapped TCF phase along the time axis shows a pulse when the TCF phase is in the ramp stage and zero otherwise. Therefore, the differentiation can emphasize the difference between the cross term and auto-term regions (Figure 5).

### 3.3. Median Filter

**Figure 6:**
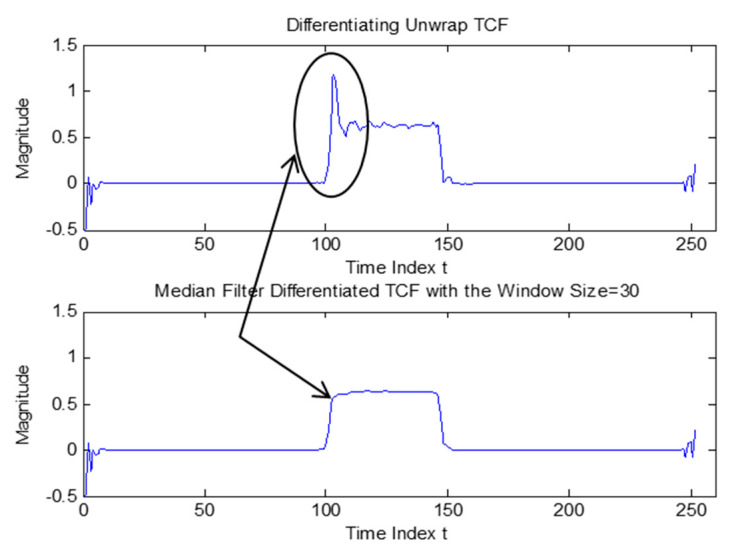
The median filter impact on pulse−like signal: top plot: original signal; bottom plot: median filter output with median filter of length 30. Reprinted with permission from Ref. [7]. Copyright 2008, Y-.P. Cheng

In Figure 6 above, the small variations within the expected constant regions or the linear ramp region are high-frequency noises that emerge from differentiation processing. The median filter is designed to remove short-term spike distortions while maintaining long-term signal trends, which can be used to smooth out the discontinuities [11]. Median filter, a non-linear filter, input data are sorted by increasing values and the middle point is picked as the filter output so that the median filter is often used to remove outliers or short discontinuities. Thus, the short-term distortions can be discarded by selecting an opposite length of the median filter. The small oscillations on the pulse disappeared and did not affect the square pulse shape of the 50-point wide because the length of the median filter is 30, which is shorter than the pulse width, as shown in Figure 6.

### 3.4. Wavelet Analysis

To feature TCF cross term after derivative operation, wavelet analysis was introduced to extract the discontinuity transition in the data. Digital wavelet transfer (DWT) analysis was discussed and the first-order Daubechies wavelet (db1) was applied to extract the TCF phase discontinuities in the study.

**Figure 7:**
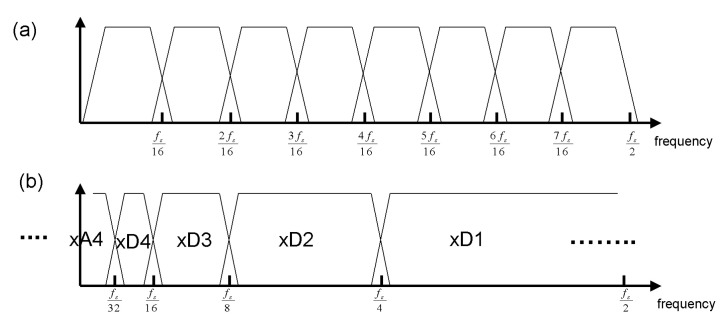
(**a**) Filter bank of STFT with constant bandwidth f_s_/6. (**b**) Four-level DWT with wide filter bandwidth f_s_/4 at the first level decomposition, while narrow bandwidth f_s_/32 filter at the fourth level. Adapted with permission from Ref. [12]. Copyright 2003, R.Cristi.

Digital wavelet transfer (DWT) is a bank of filers with half bandwidth in each further decomposition level, while a short-time Fourier transform (STFT) operated with constant bandwidth. This decomposition could be shown as the structure of the decomposition tree, as shown in Figure 7a. DWT has variable partitioning of time-frequency, leading to partitioning along the frequency (Figure 7) for four-level decomposition, as shown in Figure 7b.

In discrete wavelet transfer (DWT), the filter applied in each decomposition level, was set as 1/2 of the prior level of coefficients. The detail coefficient filter banks were illustrated in Figure 7b and the sampling frequency (fs) of the filter in each decomposition level was 1/2 of the prior level. For example, the fs of xD1 = 1/2* fs/2 = 1/4*fs, and fs of xD2 = 1/2 *xD1 = 1/8* fs. It turned out a 1/2 fs filer followed by a factor-of-2 downsampler in each decomposition level, removing the aliasing signal just right and without keeping the redundant data amount with the exceeding sample rate in the prior level.

**Figure 8:**
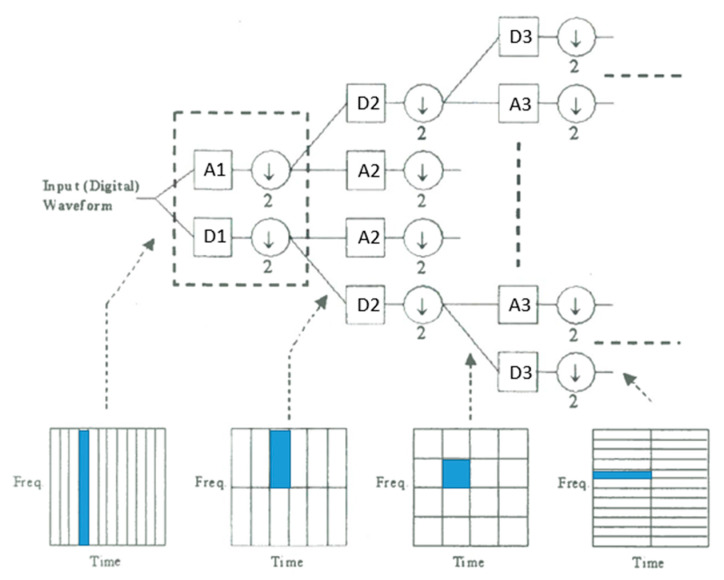
Four-level decomposition tree of the wavelet transform. The low-level decomposition (**left** part) contained low-frequency resolution and high time difference resolution, while the high-level decomposition (**right** part) had a high resolution in frequency and coarse time analysis. Reprinted with permission from Ref. [13]. Copyright 2016, Y-.P. Cheng.

Figure 8 illustrated a four-level DWT decomposition, and frequency resolution was proportional to level of decomposition while time resolution degraded accordingly. The input signal was decomposed into detail coefficients, denoted as D, and approximation coefficients, denoted as A, which contain the high-frequency information of the signal and low-frequency information in each level of decomposition. Note that detail and approximation coefficients were derived from high-pass and low-pass filters and down-sampled by a factor of 2 as bandwidth had also been decreased by that amount.

**Figure 9:**
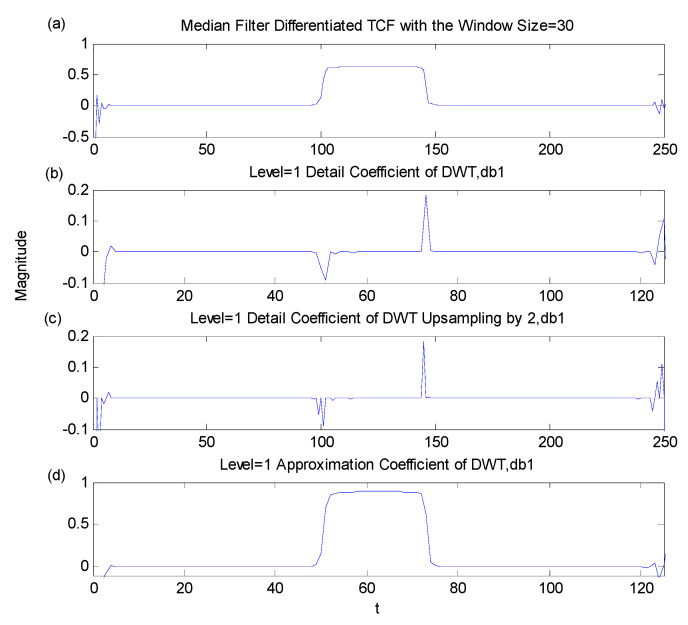
Application of the DWT to the processed TCF phase at a fixed lag τ; (**a**) unwrapped TCF phase, (**b**) detail coefficients, first−level DWT, (**c**) detail coefficients after up-sampling by 2, (**d**) approximation coefficients, first−level DWT of (**a**).

Figure 9a exhibits a typical TCF phase plot obtained for a fixed lag value τ = 25 from no noise signal. A clear pulse is displayed in the trace on the TCF phase. Figure 9b shows the wavelet coefficients after first-level DWT processing. Note the wavelet coefficients clearly identified the rising and falling edges of the unwrapped TCF phase in Figure 9a as the spikes indicated the discontinuities. For maintaining the same dimension as the original TCF plot, a 2-bit up-sampling was applied to the DWT output in this research and the results were shown in Figure 9c. Approximation coefficients containing the low-frequency signal information are shown in Figure 9d. Approximation coefficients were not used in this detection scheme.

## 4. Morphological Operations

Section 3 showed the enhancement processes that emphasized the discontinuities present in the TCF phase, which were later used to identify agile frequency timing information. This section introduces the image processing methods used and created in this research to resolve hopping time signature. First, edge detection schemes are discussed for modifying the TCF graph for image processing feasible type. Next, image denoise operations are applied to enhance meaningful TCF image signatures. Last, Hough transform was described to estimate hopping timing and complete the detection scheme.

### 4.1. Cross-Term Edge Finding

Edge detection is an intense research topic in image processing because the edge signal includes a significant amount of information. A properly derived edge figure also drops the complexity of raw data as a result of improvement in computational power capabilities. Applications can be found in manufacturing with automated categorization of parts [14], target recognition schemes for advanced driver assistance systems [15], radiology for medical applications [16], etc. For simplification of later processing, stages could cause issues because of thresholding when the grayscale or color image is transformed into a binary image.

Gradient values contain amounts of image information and catch significant image information by preserving gradient values. Thus, the fundamental concept of edge detection is based on variation in gradient magnitude and direction [17] at the region boundaries. However, the specific cross-term boundary orientation of TCF was beneficial to matched mask process in emphasis and denoise operation. A matched-filter operation was designed for an efficient edging process to extract TCF information in this study.

#### 4.1.1. Sobel Operator

The energy level difference between two adjacent pixels along the axis can be calculated by the first-order gradient. Equations (6) and (7) [17] derived the expression for first-order pixels energy gradient approximation *g_x_*(*x*,*y*) and *g_y_*(*x*,*y*) along the *x*-axis and *y*-axis, respectively, from the original values *p*(*x*,*y*).

(6) $$g_{x}(x,y)={\frac{\partial p}{\partial x}|}_{\left(x,y\right)}=p(x,y)-p(x-1,y),$$

(7) $$g_{y}(x,y)={\frac{\partial p}{\partial y}|}_{\left(x,y\right)}=p(x,y)-p(x,y-1).$$

The Sobel operator uses pixel weighting in the gradient computation to introduce smoothing. Calculation of the first-order gradient approximation of the Sobel operator along the *x*-axis is shown in Equations (8) and (9) [18]:

(8) $$g_{x}(x,y)={\frac{\partial p}{\partial x}|}_{\left(x,y\right)},$$

(9) $$\begin{array}{cc} \Rightarrow g_{x}(x,y) & =p(x+1,y-1)+2p(x+1,y)+p(x+1,y+1)\cdots \\ & -p(x-1,y-1)-2p(x-1,y)-p(x-1,y+1). \end{array}$$

Similarly, calculation of the first-order gradient approximation of the *y*-axis is shown in Equations (10) and (11):

(10) $${g_{y}(x,y)=\frac{\partial p}{\partial y}|}_{\left(x,y\right)},$$

(11) $$\begin{array}{cc} \Rightarrow g_{y}(x,y)= & p(x-1,y+1)+2p(x,y+1)+p(x+1,y+1)+\cdots \\ & -p(x-1,y-1)-2p(x,y-1)-p(x+1,y-1). \end{array}$$

The Sobel operator applied two 3 × 3 kernels [19], one for calculating the gradient along horizontal direction [20] as Equation (12)

(12) $$gx=\left[\begin{array}{ccc} -1 & 0 & 1\\ -2 & 0 & 2\\ 1 & 0 & 1 \end{array}\right]*p\left(x,y\right),$$

while another one processed difference in vertical direction and defined it as follows [20]:

(13) $$gy=\left[\begin{array}{ccc} 1 & 2 & 1\\ 0 & 0 & 0\\ -1 & -2 & -1 \end{array}\right]*p\left(x,y\right).$$

Note that final edge pixels were screened by thresholding the processed gradient values.

#### 4.1.2. Robert Operator

For the image focusing on diagonal oriented pattern, a column-wise and row-wise aligned kernel edge operator, such as Sobel, may introduce redundant and misleading pixels as the kernel weighting based on a squared-contour mask. Robert operator, on the other hand, calculated the gradient intensity by two diagonally aligned 2 × 2 kernels, which distinguish the diagonal edges efficiently. The *x*-axis gradient was defined as [21]:

(14) $$gx=\left[\begin{array}{cc} 1 & 0\\ 0 & -1 \end{array}\right]*p\left(x,y\right),$$

while the *y*-axis gradient was defined as

(15) $$gy=\left[\begin{array}{cc} 0 & 1\\ -1 & 0 \end{array}\right]*p\left(x,y\right),$$

the magnitude of gradient was calculated as follows:

(16) $$\left|G\right|=\sqrt{G_{x}^{2}+G_{y}^{2}}.$$

Robert edge operator introduced fewer aliases in processing image borders than matched orientation for diagonal kernels.

**Figure 10:**
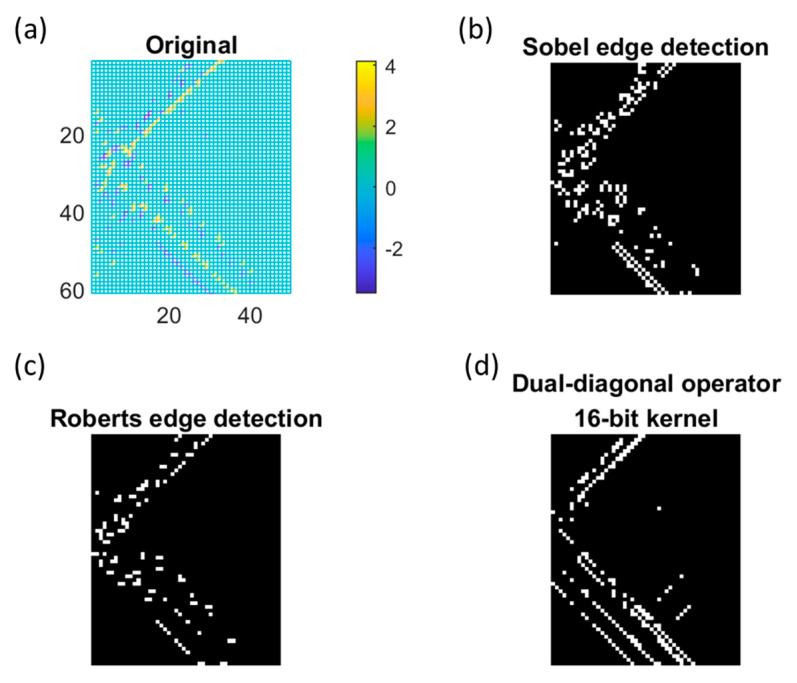
(**a**)A 2D image of noisy TCF phase after wrapped operation. The results of (**b**) Sobel edge detection, (**c**) Roberts edge detection, and the (**d**) dual-diagonal operator with 16−bit kernel.

Figure 10a was a noisy unwrapped TCF phase image containing a discontinuous cross phase term with a right triangle vertex. The cross-term boundaries were diagonally aligned at ±45 degrees. Sobel operation was applied and shown in Figure 10b. Edge detection introduced a cluster of pixels as the squared-contour kernel enlarged the diagonally aligned raw image into a cluster. Such aliasing degraded precision and increased false alarm in the timing estimation scheme in this study Figure 10c showed the results of Robert operator. The retained edge pixels were aligned diagonally along the raw input image without distorting the alignment.

#### 4.1.3. Dual-Diagonal Operator

The kernel of Robert operator fit better to diagonal aligned image than other squared-contour operators. However, the 2 × 2 kernels were too crude to process uneven boundaries properly, such as the TCF in low SNR scenarios. To emphasize the V-shaped signature of TCF in this study, dual-diagonal operator (DDO) matching the pattern with a V-shaped kernel was introduced in this research to mend the discontinue gradient along the timing signature from the boundaries of TCF and suppress the influence from mismatching distortion. Matched filtering operation of the signal by a V-shaped kernel of 45 degrees was applied for enhancing the specific ±45-degree-oriented character and denoise was processed to a mismatched pattern.

A schematic 45-degree V-mask with a length of four-bit kernel was defined as follows:

(17) $$V=\left[\begin{array}{cccc} 0 & 0 & 0 & 1\\ 0 & 0 & 1 & 0\\ 0 & 1 & 0 & 0\\ 1 & 0 & 0 & 0\\ 0 & 1 & 0 & 0\\ 0 & 0 & 1 & 0\\ 0 & 0 & 0 & 1 \end{array}\right].$$

The 2D convolution formulation was defined as follows:

(18) $$R\left(i,j\right)=\underset{m}{\sum }\underset{n}{\sum }V\left(m,n\right)TCF\left(i-m\right)\left(j-n\right),$$

where V is the 45-degree V-kernel, TCF is the absolute value of the unwrapped TCF data, and i, j are the indices of *x*-axis, *y*-axis in a range of the kernel dimension accordingly.

**Figure 11:**
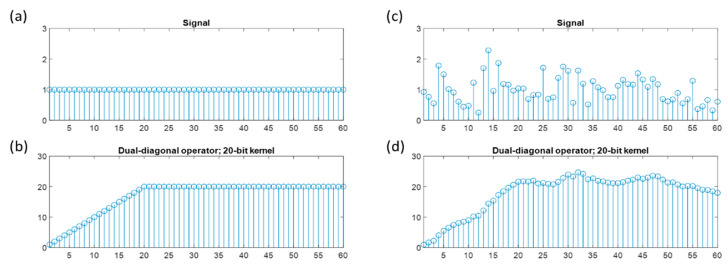
Illustrative matched filtering example of one kernel of dual-diagonal operator (DDO). (**a**) Noise-free signal with amplitude equal to one; (**b**) matched filtering by a 20-bit linear kernel with processing gain equal to the kernel size; (**c**) a noisy signal showed the distortion in amplitude; (**d**) matched filtering output of (**c**) showed the noise durability by earning the processing gain through one-dimensional DDO.

Convolution was a multiply/accumulate operation. A noise-free DC signal was shown in Figure 11a with amplitude equal to one. After convolution operation of 20-bit mask kernel, the signal increased up to twenty-fold of the original data amplitude when x index was larger than kernel length for a best match gain, as shown in Figure 11b. Figure 11c showed a noisy signal scenario. A noise-resistant signal processed by convolution was shown in Figure 11d. Matched filter gain was retained by accumulating the amplitude of the signal within the kernel, and matched filtering convolution output increased significantly compared to the original noisy signal. This matched filtering technique was advantageous to identify the signal location and enhance the noise durability of the systems [22].

Following the convolution operation, an adaptive threshold, generated by a median filter enclosed by a DDO kernel, was applied to the final edge detection. The DDO median operation was defined as follows:

(19) $${Median\;of\;DDO}_{TCF},\;M=\{\begin{array}{c} DDO\text{\_}Sorted\left[\frac{(k_{u}+k_{b})+1}{2}\right]\;\;,if\;k_{u}+k_{b}\;is\;odd,\\ \frac{DDO\text{\_}Sorted\left[\frac{k_{u}+k_{b}}{2}\right]+DDO\text{\_}Sorted\left[\frac{k_{u}+k_{b}}{2}+1\right]}{2},\;if\;k_{u}+k_{b}\;is\;even, \end{array}$$

where *DDO_TCF*(*k_u_*, *k_b_*) was the TCF processed by DDO convolution in the range of the V-shaped kernel in the size of ku-bit and kb-bit to the upper and bottom branches, respectively. *DDO_Sorted*[*k*] was the sorted data vector enclosed within *DDO_TCF*(*k_u_*, *k_b_*), and the threshold baseline was chosen as a median value out of the vector. The threshold was set according to the multiple of the baseline median value of the convolution gradient within the kernel. When the convolution gradient was over the threshold, the pixel was retained. The median-based threshold can avoid convolution results with skew and heavy-tailed [23].

A comparison example of DDO applied on TCF was shown in Figure 10d. The fractional segments with uneven gradient of TCF were mended without deforming the diagonal boundaries after the edge finding process. Note that the disoriented signal on the TCF might not be retained after the threshold applied due to the scarce integration gain earned by the DDO compared with the matched pattern scenarios.

### 4.2. Denoise Masking Design

However, solely relying on edge detection schemes might still retain much unavailable information from background noise or defect shapes problems from the gradient level threshold steps. Thus, specific morphological processing was applied to enhance the matched pattern information and to make it more useful for later stages. Next, the denoise operations based on the morphology were discussed.

#### 4.2.1. Dilation

**Figure 12:**
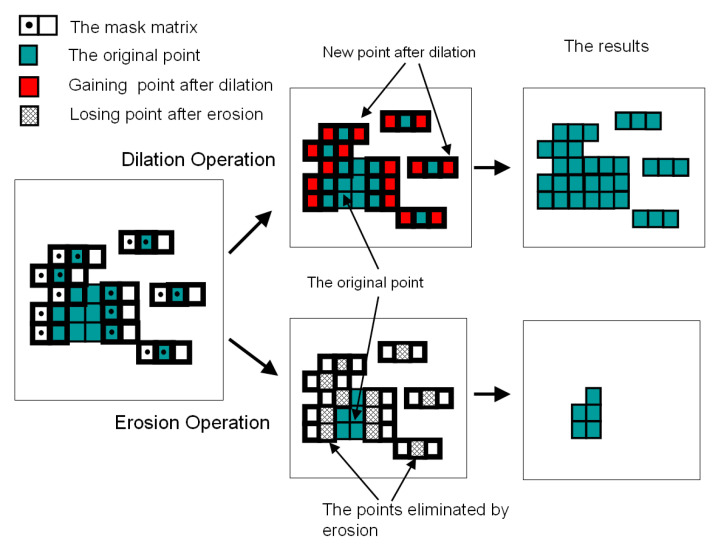
Morphological denoise operation by 2−bit landscape kernel. The top branch was the dilation operation for mending fractional pixels; the bottom branch showed the erosion operation removing mismatched pixels. Adapted from [24].

Dilation is a “union” operation between the object and a kernel with a designated alignment fitting a specific pixel arrangement. The schematic diagram of the dilation operation and the “expended” result were shown in the top branch plot of Figure 12. The kernels enlarged the original object by filling in small intrusions found in uneven blobs, resulting in an image with fewer fractional sections. Note that the dilation operation did not add any information to the image when there was no original pixel enclosed by the kernel.

#### 4.2.2. Erosion

In contrast to the dilation operation expanding pixels with the trend of matching the kernel contour, erosion processing can remove independent or small speckles from the image. The erosion operation is an intersection operation between the object and a kernel matrix, as shown in the bottom branch of Figure 12. The erosion operation cleans isolated pixels because pixels grouping was not enclosed by the kernel matrix completely.

Therefore, the patterns matching the contour of the kernel had a higher chance to be retained and those mismatched patterns were the target to be removed in this denoise process. Diagonal aligned kernels were applied in this research to retain the proper oriented figure from TCF and suppressed distorting pixels.

When the signal characteristic matched the kernel, the processing kept the matched pixels and decayed the mismatched blobs. A 3-bit diagonal kernel as the mask was defined as:

(20) $$\left[\begin{array}{ccc} 1 & 0 & 0\\ 0 & 1 & 0\\ 0 & 0 & 1 \end{array}\right]$$

### 4.3. Line Segments Resolving

After a series of emphasizing and denoise processes were discussed in Section 3 and prior to Section 4.2, the timing signature of FHSS was revealed and modified as diagonal line segments, which indicate the timing information at the time index intersection. Line equation resolving methods were introduced next as the final step of the estimation scheme.

#### Hough Transform

Hough transform is widely applied to image analysis applications, especially in detection of lines, curves, or circles [25]. Hough detection was applied for finding the apex of the diagonal lines of TCF phase region boundaries, which is the location of hopping time in this study.

**Figure 13:**
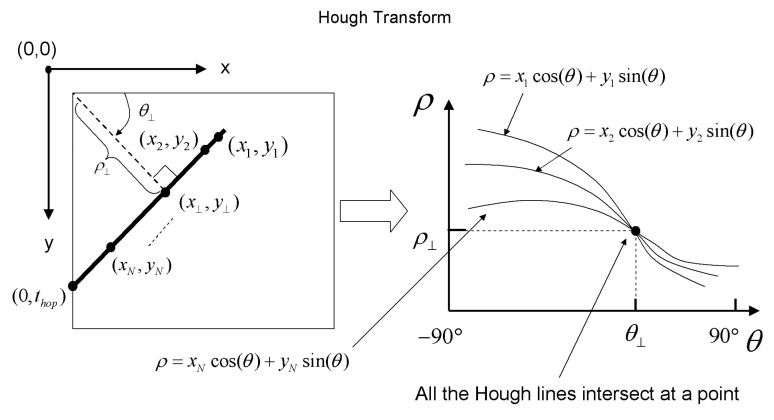
Hough transform (**left** plot) and Hough line equations (**right** plot). Adapted from [24].

The two parameters of the Hough transform are *θ*_⊥_ and *ρ*_⊥_, which are transformed from the line in the x-y plane. They can represent a line (shown in Figure 13 as the dotted line) orthogonal to the line of interest and pass the origin. Parameter *ρ*_⊥_ is the shortest distance from the origin to the line of interest, and *θ*_⊥_ is the included angle between the *x*-axis and the line of interest, as shown in Figure 13. Thus, the relation of (*x_i_*, *y_i_*) and (*ρ*, *θ*) may be written as:

(21) $$y_{i}=-\frac{\cos(\theta )}{\sin(\theta )}x_{i}+\frac{\rho }{\sin(\theta )},$$

which could be rewritten as:

(22) $$\rho =x_{i}\cos\theta +y_{i}\sin\theta .$$

Equation (22) corresponds to a sinusoidal curve in the (*ρ*, *θ*) plane. In Figure 13, all points on the line of interest correspond to sinusoidal curves in the (*ρ*, *θ*) plane and intersect at the specific value (*ρ*_⊥_, *θ*_⊥_). Therefore, the points on the line of interest in the original image plane can infer the specific point (*ρ*_⊥_, *θ*_⊥_) in the (*ρ*, *θ*) plane. In contrast, the line can be derived according to parameter (*ρ*_⊥_, *θ*_⊥_). The intersection (*x*_⊥_, *y*_⊥_) is the shortest distance (*ρ*) from the origin to the line of interest, which can be calculated by:

(23) $$\{\begin{array}{c} x_{⊥}=\rho_{⊥}\cos\theta_{⊥}\\ y_{⊥}=\rho_{⊥}\sin\theta_{⊥} \end{array}.$$

The slope of the line in the x-y plane was calculated by:

(24) $$\mathrm{line}\;\mathrm{slope}=\frac{y-y_{⊥}}{x-x_{⊥}}.$$

Note the line slope value could be also expressed in terms of the angle *θ*_⊥_ as tan(90° − *θ*_⊥_); then, the equation was presented as:

(25) $$\tan(90{}^{\circ}-\theta_{⊥})=\frac{y-\rho_{⊥}\sin\theta_{⊥}}{x-\rho_{⊥}\cos\theta_{⊥}}.$$

Furthermore, note the intersection, hopping time information *t_hop_*, is on the *y*-axis in the TCF phase plot, as shown in Figure 2. Therefore, the intersection point *t_hop_* could be determined by setting x = 0 in Equation (26) as follows:

(26) $$t_{hop}=y=\rho_{⊥}(\sin\theta_{⊥}-\tan(90{}^{\circ}-\theta_{⊥})\cos\theta_{⊥}).$$

Therefore, *ρ* and *θ* of the Hough transform could judge the line regarding whether it has the same intercepted points on the plot [26,27].

## 5. Detection Simulation

This section presented the processes in these two phases and the overall detection performance evaluations.

### 5.1. Simulation Overview

To evaluate the FHSS estimation algorithms proposed in the research, one thousand Monte Carlo experiments were carried out with signal-to-noise ratio (SNR) sweeping in the range of −3 dB to 15 dB to tally the successful estimation trials, and the results are presented as the probability of detection (Pd). In addition to Pd evaluation, one thousand trials of constant frequency Monte Carlo experiments on the estimation scheme were practiced to evaluate the probability of false detection (Pfa) for study reference.

### 5.2. Phase 1: Timing Signature Enhancement

The processing steps conducted in phase 1 of the estimation scheme to enhance the timing signature of the FHSS signal, which was the left group of the scheme flow chart in Figure 3, were described in this section.

#### 5.2.1. FHSS Signal Generation

The analytical frequency agile signal with one frequency transition was conducted in the experiment and defined as follows:

(27) $$x\left(t\right)=\cos\left(2\pi f_{n}\times \frac{T_{n}}{f_{s}}t\right),\;n=1,2,$$

where *f_s_* was the sampling frequency, *f*_1_ and *f*_2_ are the symbol frequencies, and *T*_1_ and *T*_2_ are the symbol pulse signal durations, respectively.

The sampling frequency was set *f_s_* = 150 Hz in the experiment with first signal frequency *f*_1_ = 15 Hz and second signal frequency *f*_2_ = 45 Hz. Signal duration *T*_1_ = 1.13 s, *T*_2_ = 0.87 s. The total number of samples in the FH frame was equal to (*T*_1_ + *T*_2_) × *f_s_* = 2 × 150 = 300, and the frequency change occurred at sample *t_hop_* = *T*_1_ × *f_s_* = 1.13 × 300 = 130. Note, a fixed timing index was for only while the overall timing signature extraction was under blind estimation with noise variation.

For a frequency-agility-free experiment, set *f*_1_ = *f*_2_ for generating a single tone signal in Pfa evaluating simulation.

#### 5.2.2. Temporal Correlation Function (TCF) Computation

The TCF was computed following Equation (4) with the analytical FH signal defined in Equation (27). An illustrative TCF was shown in Figure 2 with the frequency changing at t-index = 130, which was the configuration applied in the evaluation process.

#### 5.2.3. TCF Phase Region Edges Enhancement

To enhance the timing signature from the TCF phase term, a series of t-axis-wise processes were executed in sequence.

(1) TCF phase unwrapping; also refer to Section 3.1.
(2) Differentiation of the unwrapped TCF; refer to Section 3.2.
(3) Median filtering differentiated TCF; refer to Section 3.3.
(4) The first order coefficients of DWT; refer to Section 3.4.

### 5.3. Phase 2: Morphological Operations

The cross-term trajectories of the TCF phase term were emphasized at the phase 1 stage and the timing signature was further processed and extracted by the phase 2 image morphological process schemes, which were illustrated at the right-side flowchart of Figure 3. In this phase, TCF was processed by (1) edge finding, (2) denoise, and (3) line segments resolving, three functions based on the pattern-matching operation.

#### 5.3.1. DDO Edge Finding

**Figure 14:**
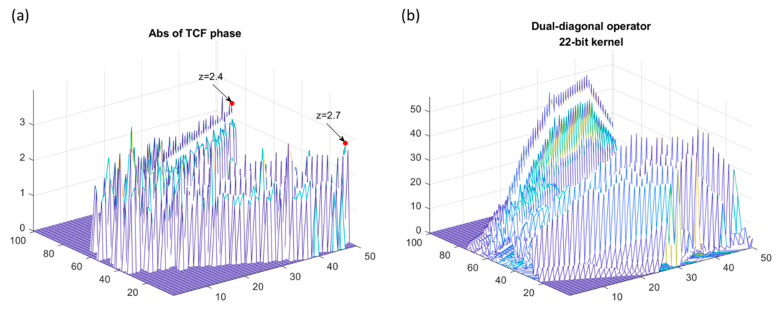
TCF was processed by a dual-diagonal operator. (**a**) An SNR = 9 dB noisy TCF phase term processed by DWT with amplitude around 2.4. (**b**) Post-DDO processed (**a**) with a 22-bit kernel; the matched pattern amplitude increased to 50.

Because the DDO kernel was specified as a 45-degree V-shaped kernel, the meaningful character of TCF was emphasized by matched filtering operation and the non-pattern-matched disoriented signal tended to be muted. Figure 14a was a preprocessed noisy TCF signal with SNR = 9 dB. The amplitude of the cross-term trajectory was around 2.4, surrounded by spurious distortions. Figure 14b demonstrated the post-DDO processed TCF signal amplitude was accumulated up to 50 by the 22-bit V-shaped kernel, which was the multiple of the original signal amplitude enclosed within the kernel. Note that the disoriented mismatched signal did not earn gain significantly compared to the matched-pattern signal.

**Figure 15:**
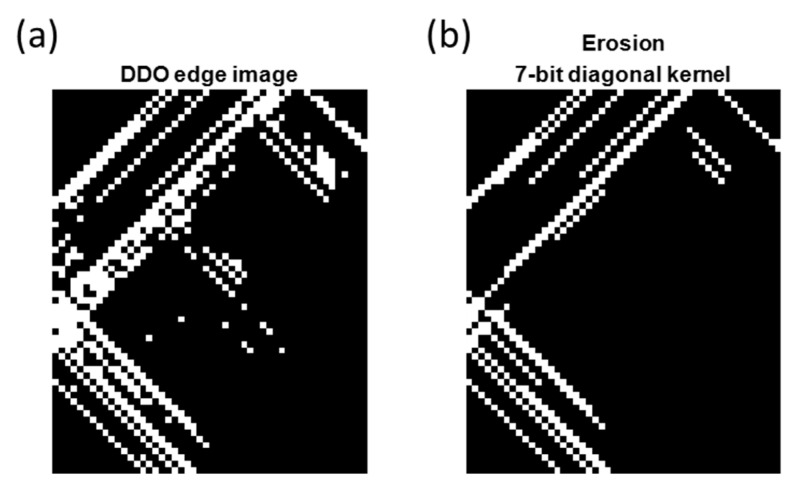
Erosion operation: (**a**) DDO edge detection; (**b**) erosion operator with 7-bit diagonal kernel.

To complete the edge-finding process, a dynamic threshold was determined by the median value within the process kernel multiplied by two. The post-DDO-processed TCF edge bitmap was shown in Figure 15a. The majority of the timing signature was captured and fulfilled accompanied by some potential distortion segments, which require the next stage, denoise process, to purify.

#### 5.3.2. Erosion Denoise Process

A 7-bit diagonal kernel erosion operator processed the Figure 15a bitmap in ±45° orientation, respectively, in this study and the denoise bitmap, as shown in Figure 15b. The erosion kernel removed the less consistent segments from the TCF bitmap, which was most likely carrying distortion information.

#### 5.3.3. Line Segments Resolving

**Figure 16:**
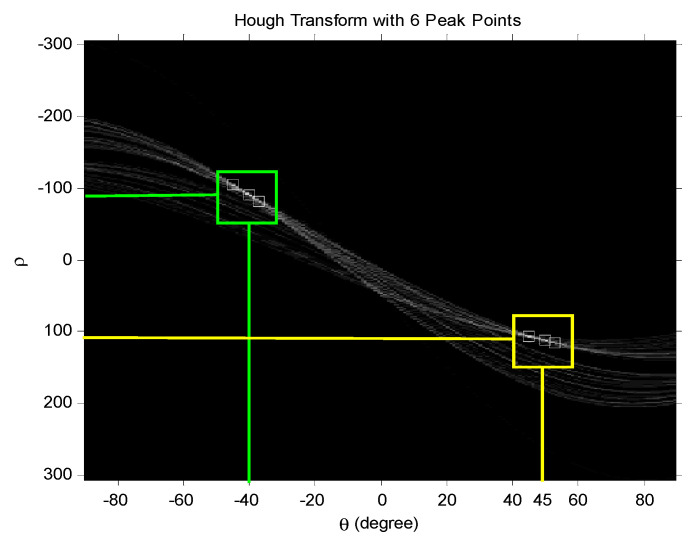
Hough transform lines with 6 peak points.

After the prior denoising process, the retained line segments were preserved with abundant meaningful timing signatures. Hough detection was applied to resolve the estimated hop time in the final stage. Figure 16 was the Hough transform plane resolving the line segment in Figure 15b, and there were six peaks kept in the scheme. Each *ρ* and *θ* corresponded to an estimated line equation represented in the Hough plane.

Recall that the lines of interest in the TCF plane were expected to be ±45° orientations. Therefore, those line alignments not matching these orientations were discarded from the final *t_hop_* estimation process.

### 5.4. Different Configuration Option Results

**Figure 17:**
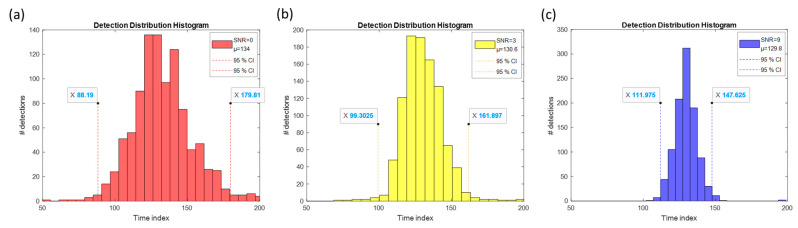
Histogram showing the distributions of estimated results in (**a**) SNR = 0 dB, (**b**) SNR = 3 dB, and (**c**) SNR = 9 dB. The mean and 95% confidence interval are shown.

The distributions of Hough detection results were performed in one thousand trials, as shown in Figure 17. The mean of the results was 134, 130.6, and 129.8 as the true *t_hop_* was set as 130 in the experiment. The 95% confidence interval was 45.8, 31.3, and 17.8 at SNR 0, 3, and 9 dB in (a), (b), and (c) subplots, respectively. As the SNR increased, the variance in the estimated decreased *t_hop_* and the trend went reversely otherwise. Note that the mean estimated *t_hop_* was still close to the true setup value 130, which proves the estimation scheme yielded truthful results at SNR = 0 dB and the estimation errors were inherently introduced by variation in the AWGN. The tolerance of the possibility of detection referred to the 95% confidence interval at SNR 3 dB in this study for performance comparison of different configurations.

#### 5.4.1. Configuration Options

Kernel size and thresholding were two primary parameter configurations of DDO that would dominate the overall timing estimation performance in this paper. A larger kernel and lower threshold of DDO enclose more signals for the accumulation process; however, they also took misleading noise information into account. In contrast, an insufficient size of kernel or an over-selected threshold degraded the performance in matched-pattern discrimination due to the scarce data considered.

To acquire a proper configuration of this FH timing detection scheme, iteration of kernel size and multiple thresholds were performed regarding the results of Pd and Pfa. The proper configuration was chosen by the best performance in Pd and with Pfa under control.

**Figure 18:**
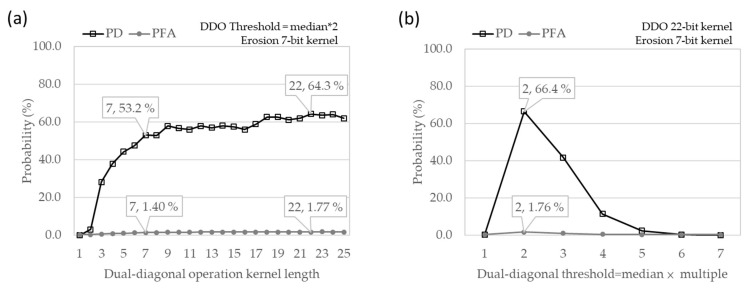
The sweeping of the (**a**) dual-diagonal operator window size and the (**b**) dual-diagonal operator threshold of multiples of the median at SNR = −3 dB.

In Figure 18a, DDO kernel size had been tested from 1-bit up to 25-bit by a one-thousand-trial baseline signal set with SNR = −3 dB. Pd rose significantly with kernel size larger than 7-bit and Pfa less than 1.4% and down to 0.33%. As a result, 22-bit kernel performs a high Pd = 64.3% with Pfa = 1.77%.

In Figure 18b, the multiples of the median for thresholding in DDO with a 22-bit kernel were tested. The multiple was swept from one to seven and the Pd had the best performance at two, which was the configuration practiced in the research.

#### 5.4.2. Performance Evaluation

The overall performance of the denoise processes and the decision algorithms introduced in this study was displayed in Table 1. The result showed a meaningful detection Pd as 62.4% when SNR was down to −3 dB, and the scheme performed with high accuracy when SNR was 0 dB and above. Note that the false alarm was controlled under 1.79% in the applicable SNR range in this study, which indicates that the morphological matching process controlled and screened out misleading distortion from the estimation procedure and provided robust noise duration to the system.

TCF-based frequency agile signal timing detection schemes had been previously studied in one-dimensional data analysis with thresholding by a multiple of the variance of processed TCF [5,6]. Even with dynamic thresholding attempting to optimize the decision rate, without taking the inherently two-dimensional TCF signature into account, the one-dimensional (1D) process failed to suppress false detection in high noise interference scenarios. The performance comparison of 1D procedures and this two-dimensional (2D) morphological process are listed in Table 2, columns 1, 2, and 3, respectively. The 1D processes seemed to perform stably in Pd. Nevertheless, the Pfa was more than 30% in the noisy scenarios, leading to untrustworthy Pd and degrading efficiency significantly for frequency analysis, with enormous false estimated timing information.

## 6. Conclusions and Discussion

An efficient FH signal timing estimation scheme benefits not only ECCM application for follow-up of the FH transmitter attempt but radio channel management as well. Frequency channel clearance is essential for all radio appliances. An efficient FH detection algorithm can ensure communication channel quality in the frequency domain by instantly monitoring the occupied radio frequency nearby.

In this study, inspired by the Roberts edge operator, which was designed for 45 and/or 135 degrees orientation detection [28], a novel dual-diagonal operator was proposed for the advantage of diagonal-figure-edge-finding application. A V-shaped kernel was beneficial to extract the timing signature from TCF through a matched filtering operation to accumulate the amplitude of the associated signal and an adaptive thresholding mechanism via a median filter. Both procedures were executed by the DDO kernel.

Because the characteristic TCF signal held a 45-degree angle composed of two diagonal lines, Sobel’s and other mismatched kernel operators were not suitable for this application. Through comparison of the Sobel and Robert operators, the DDO proposed in this research proved that this morphological matching scheme had significantly improved high Pd acquisition and steady Pfa control as well. A follow-up diagonal erosion operator also took part in the denoise process and contributed to Pfa suppression.

Through comprehensive Monte Carlo simulation to evaluate the performance of the detection scheme, the overall FH timing estimation had a robust detecting Pd of more than 90.1% when SNR was 0 dB and higher. The Pd reached nearly 100% when SNR was 3 dB and above. False detection rate (Pfa in this paper) was consistently suppressed under 1.8% even in intensity noise scenarios as the SNR decreased to −3 dB, regarding which Pfa significantly increased in previous research. The improvement was owing to the proposed morphological matched filtering scheme, which was demonstrated to be capable of eliminating misleading information and accumulating data from appropriate patterns.

The Pfa was not completely eliminated as the SNR rose in this scheme due to the TCF phase being sensitive to noise distortion and having the chance to produce a V-shaped pattern. These false signatures carrying misleading information could be caught by the schemes. Nevertheless, the majority purpose for the FH timing estimation scheme is to robustly resolve timing information in noisy information with minor Pfa under control. A false timing report does result in misleading frequency estimation as the signal was sampled at this time section. However, missing HF timing detection results in missing signal sampling time for frequency detection purposes.

Due to the sensitive phase response of TCF in an intensity noise environment, which results in a false V-shaped pattern to mislead the morphological resolving process in this study, improving the timing signature purity in phase 1 is a possible future research direction. There are two recommended directions:

(1) Developing a phase-stable form of TCF to ensure purity of timing signature raw data.

(2) Enhancing the timing signature from the discontinued TCF phase term. Different order coefficients of DWT are candidates to study.

## Figures and Tables

**Table 1:** The Pd and Pfa performance of the overall FHSS signal timing estimation scheme.

SNR (dB)	−3	0	3	6	9	12	15
PD (%)	62.4	90.1	97.0	98.7	99.4	99.8	99.9
PFA (%)	1.77	1.79	1.76	1.70	1.54	1.36	1.22

**Table 2:** Pd and Pfa percentile comparison of two literature reviewing studies with the current proposed detection scheme.

	MP Fargues. et al. [5]		M Sirotiya. et al. [6]		This Research	
SNR	PD	PFA	PD	PFA	PD	PFA
−3	69.27	33.33	85.8	33.33	62.4	1.77
0	81.29	35.29	98.2	35.29	90.1	1.79
3	89.53	19.61	99.6	19.61	97.0	1.76
6	98.44	15.69	100	15.69	98.7	1.70
9	98.66	1.96	100	1.96	99.4	1.54
12	100	0	100	0	99.9	1.22

## Data Availability

Data sharing is not applicable to this article as no datasets were generated or analyzed during the current study.
